# The Influence of the Variation in Sepsis Rate between Neonatal Intensive Care Units on Neonatal Outcomes in Very-Low-Birth-Weight Infants

**DOI:** 10.1038/s41598-020-63762-6

**Published:** 2020-04-21

**Authors:** Tae-Jung Sung, Jin A. Sohn, Sohee Oh, Jin A. Lee

**Affiliations:** 1 Department of Pediatrics, Hallym University Medical Center kangnam Sacred Heart Hospital, Seoul, South Korea; 2 Department of Pediatrics, Seoul National University College of Medicine, Seoul, South Korea; 3 Department of Pediatrics, SMG-SNU Boramae Medical Center, Seoul, South Korea; 4 Department of Biostatistics, SMG-SNU Boramae Medical Center, Seoul, South Korea

**Keywords:** Neonatal sepsis, Neonatology

## Abstract

Sepsis is commonly known to affect neonatal outcomes. We assessed how much center-to-center variability of the sepsis rate affects the outcomes of very-low-birth-weight infants (VLBWIs). 7,493 VLBWIs registered in the Korean Neonatal Network from 2013 to 2016 were classified into three groups according to the sepsis rate: low sepsis group (LS) < 25^th^ percentile versus intermediate sepsis group (IS) 25^th^–75^th^ versus high sepsis group (HS) ≥ 75^th^. The incidence density of sepsis for the LS, IS, and HS groups were 1.17, 3.17, and 8.88 cases/1,000 person-days. After propensity score matching was done for multiple antenatal and perinatal factors, the odds ratio of death, moderate to severe bronchopulmonary dysplasia and/or death, periventricular leukomalacia, and survival without major morbidities for the HS group were 2.0 (95% confidence interval 1.4–2.8), 1.9 (1.5–2.4), 1.5 (1.1–2.3) and 0.7 (0.5–0.8) when compared with the IS group, and 2.2 (1.6–3.2), 2.3 (1.8–2.9), 2.0 (1.3–2.9), and 0.7 (0.6–0.9) when compared with the LS group. There were no significant differences in those outcomes between the LS and IS groups. Hence, nationwide quality improvements to control the sepsis rate especially in units with a high sepsis rate will be helpful to improve the outcomes of VLBWIs.

## Introduction

Sepsis is known to be associated with increased length of stay, mortality and morbidities of very-low-birth-weight (VLBW) preterm infants including bronchopulmonary dysplasia (BPD) and long-term neurodevelopmental impairment^1–5^. Nowadays, many efforts to reduce sepsis in the neonatal intensive care unit (NICU) are ongoing by nationwide quality improvement interventions including vigorous hand washing, catheter bundle management, increasing the diagnostic accuracy of sepsis and antibiotic stewardship^6^. However, it is still difficult to reduce the sepsis incidence to zero in NICU care because of the immature immunity systems of preterm infants and the different environments in each hospital which cannot be changed in a short time. Although the survival rate of VLBW infants is increasing by advances in neonatal care, the rate of sepsis is still higher in immature and smaller preterm infants.

There have been reports suggesting unit to unit differences in neonatal outcomes^7^, and such a unit difference is derived from differences in practice^8–11^. The sepsis rate usually differs from center to center^1,9–11^. Considering the adverse effect of sepsis on neonatal outcomes in preterm infants, knowing how much different the sepsis rate is between NICU units in South Korea is important to determine the necessity of managing nationwide quality improvements to control sepsis and reduce such gaps between units.

Since 2013, almost all the NICUs in South Korea have been participating in the nationwide Korean Neonatal Network (KNN). According to an analysis of the KNN database in 2014, the sepsis rate in VLBW infants in South Korea was 21.1%, and the late-onset sepsis (LOS) rate was 19.4% with a 16.1% mortality rate^12^. Such a sepsis rate is similar to that of the Canadian Neonatal Network, however, higher than that of the Neonatal Research Network of Japan^13^. We still do not know how much the sepsis rate differs from center to center in South Korea. Moreover, we do not know whether such differences in the sepsis rate are associated with a worse neonatal outcome in VLBW infants. Thus, it is necessary to determine the real status of the unit variation in the sepsis rate or the incidence density using the KNN database before starting a nationwide intervention for quality improvement to control sepsis in NICUs in South Korea.

Hence, the aim of this study was to show how much the sepsis rate or the incidence density of sepsis is different between NICUs in South Korea and how this unit variation affects the neonatal outcomes in VLBW infants.

## Results

### Population

**Figure 1:**
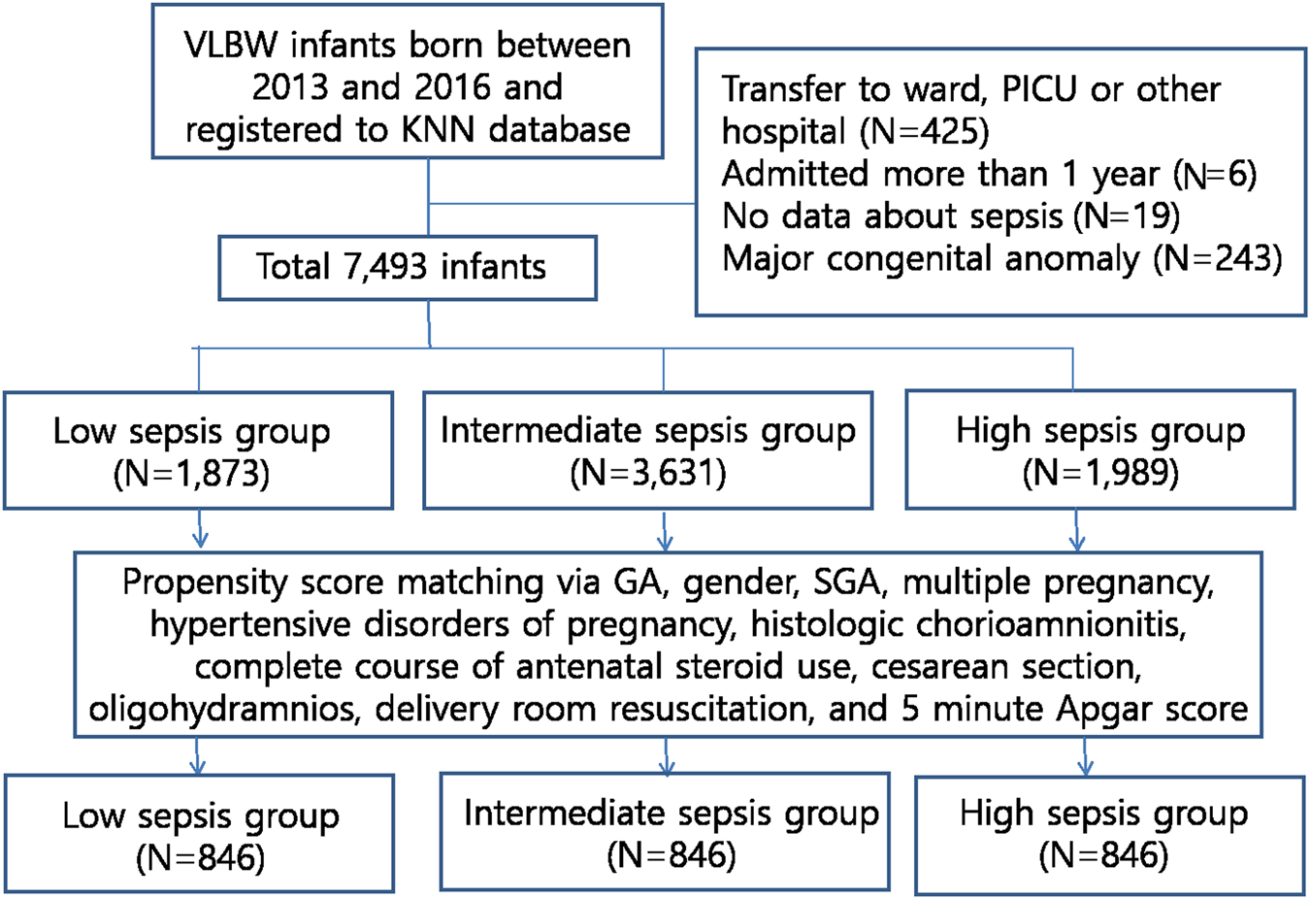
The study population. A total of 7,493 very-low-birth-weight infants born between 2013 and 2016 and registered in the Korean Neonatal Network database were included. After the propensity score matching by gestational age, gender, small for gestational age, multiple pregnancies, cesarean section, hypertensive disorders of pregnancy, histologic chorioamnionitis, oligohydramnios, complete course of antenatal steroid use, delivery room resuscitation, and 5-minute Apgar score, 846 neonates were included each in the low sepsis, intermediate sepsis, and high sepsis groups.

A total of 7,493 VLBW infants from 67 hospitals were included after the exclusion of 693 infants: 425 infants transferred to a ward, pediatric intensive care unit or other hospital; 6 infants admitted more than one year; 19 infants with no information about sepsis, and 243 infants with a major congenital anomaly. The number of infants classified as the low sepsis group (LS), intermediate sepsis group (IS), and high sepsis group (HS) was 1,873, 3,631, and 1,989 infants in each group. The number of hospitals included in the LS, IS, and HS group were 16, 35, and 16, respectively. After propensity score matching for gestational age (GA), gender, small for gestational age (SGA), multiple pregnancies, cesarean section, hypertensive disorders of pregnancy, histologic chorioamnionitis (HCA), oligohydramnios, complete course of antenatal corticosteroid (ACS) use, delivery room resuscitation, and 5 minute Apgar score, 846 infants were included in each group for the analysis (Fig. 1).

### General characteristics about sepsis

The total incidence density of sepsis in the 7,493 VLBW infants was 3.83 cases per 1,000 person-days and 1,461 infants (19.5%) experienced sepsis during NICU hospitalization. In detail, 1,393 infants (18.6%) suffered from bacterial sepsis, and 139 infants (1.9%) suffered from fungal sepsis. Additionally, 299 infants (4.0%) experienced sepsis more than one time during hospitalization. Moreover, 130 neonates (1.7%) suffered from early-onset sepsis, and 1,331 neonates (17.8%) suffered from LOS. The median value for the first postnatal day of sepsis was 15 days after birth (interquartile range 8, 28 days).

The incidence density of sepsis in each group was 1.17 cases per 1,000 person-days in the LS group, 3.17 cases per 1,000 person-days in the IS group, and 8.88 cases per 1,000 person-days in the HS group. Between the three groups, there was a significant difference in the percentage of sepsis (P = 0.001). Early-onset sepsis (EOS) was less prevalent in the LS group when compared with the IS or HS group (P = 0.002). The late-onset sepsis (LOS) rate was also significantly different between the three groups (P < 0.001), and the post-hoc analysis showed that there was also differences between the LS and IS, LS and HS, and IS and HS groups, respectively. LOS rate was highest in HS group and lowest in LS group. Additionally, the percentage of multiple episodes of sepsis was also significantly different between the three groups. There was no difference in the first postnatal day of sepsis between the three groups (Table 1).

**Table 1:** General characteristics about sepsis in the study population.

	Propensity score matching							
	Unmatched data set (N = 7,493)				Matched data set (N = 2,538)			
	Low sepsis group (N = 1,873)	Intermediate sepsis group (N = 3,631)	High sepsis group (N = 1,989)	*P*-value	Low sepsis group (N = 846)	Intermediate sepsis group (N = 846)	High sepsis group (N = 846)	*P*-value
Sepsis incidence density (cases per 1,000 person-day)	1.17	3.17	8.88	<0.001^*,**,†^	1.27	2.99	9.04	<0.001^*,**,†^
Sepsis, total, number (%)	125 (6.7%)	656 (16.9%)	721 (36.2%)	<0.001^*,**,†^	65 (7.7%)	137 (16.2%)	320 (37.8%)	<0.001^*,**,†^
Early-onset	26 (1.4%)	52 (1.4%)	52 (2.6%)	<0.001^*,**,†^	19 (2.2%)	13 (1.5%)	21 (2.5%)	<0.001^*,**,†^
Late-onset	99 (5.3%)	563 (15.5%)	669 (33.6%)		46 (5.4%)	124 (14.7%)	299 (35.3%)	
Sepsis, bacterial, number (%)	114 (6.1%)	580 (16.0%)	699 (35.1%)	<0.001^*,**,†^	60 (7.1%)	129 (15.2%)	314 (37.1%)	<0.001^*,**,†^
Sepsis, Fungal, number (%)	11 (0.6%)	63 (1.7%)	65 (3.3%)	<0.001^*,**,†^	5 (0.6%)	10 (1.2%)	23 (2.7%)	0.001^**^
Sepsis, multiple, number (%)	18 (1.0%)	112 (3.1%)	169 (8.5%)	<0.001^*,**,†^	9 (1.1%)	28 (3.3%)	74 (8.7%)	<0.001^*,**,†^
First postnatal day of sepsis(days)	14 [5,27]	15 [7,28]	15 [8,27]	0.172	13 [0, 23]	14 [7,27]	16 [9,29]	0.029^**^

Propensity score matching via gestational age, gender, small for gestational age, multiple pregnancies, cesarean section, hypertensive disorders of pregnancy, histologic chorioamnionitis, oligohydramnios, complete course of antenatal corticosteroid use, delivery room resuscitation, and 5 minute Apgar score.

Kruskal-Wallis test or chi-square test in both propensity score unmatched population and propensity score matched population.

*P < 0.05 between low sepsis group and intermediate sepsis group.

**P < 0.05 between low sepsis group and high sepsis group.

^†^P < 0.05 between intermediate sepsis group and high sepsis group.

After the propensity score matching, the incidence density of sepsis in 2,538 matched VLBW infants was 3.94 cases per 1,000 person-days, and the incidence density of sepsis in each matched group was 1.27 cases per 1,000 person-days in the LS group, 2.99 cases per 1,000 person-days in the IS group, and 9.04 cases per 1,000 person-days in the HS group. The sepsis rate was also higher in the HS group, which was mainly due to the increased incidence of LOS. The first postnatal day of sepsis was later in the HS group when compared with the LS group (P = 0.029) (Table 1).

### Differences between the three groups before the propensity score matching

#### Maternal demographics and neonatal characteristics of the three groups

GA and birthweight were higher in the LS group when compared with those of the IS and HS groups. There was no difference in the gender proportion between the three groups. Maternal hypertensive disorders of pregnancy and SGA were more frequent in the LS group when compared with those of the IS and HS groups. Less infants received a complete course of ACS in the HS group when compared with the LS and IS groups. There was no difference in the frequency of delivery room resuscitation between the three groups; however, the Apgar score at 1 and 5 minutes was significantly higher in the LS group when compared with those of the IS and HS groups (Table 2). When we compared the distribution of groups according to the mean annual number of VLBW infants registered in KNN, there was no significant differences between the LS, IS, and HS groups (Table 3). The median groups (25^th^, 75^th^ percentile) of the mean annual number of VLBW infants in the LS, IS, and HS groups were group 6 (group 4, group 6), group 6 (group 3, group 7), and group 5 (group 4, group 6).

**Table 2:** Maternal demographics and neonatal characteristics in the study population.

	Propensity score matching							
	Unmatched data set (N = 7,493)				Matched data set (N = 2,538)			
	Low sepsis group (N = 1,873)	Intermediate sepsis group (N = 3,631)	High sepsis group (N = 1,989)	P-value	Low sepsis group (N = 846)	Intermediate sepsis group (N = 846)	High sepsis group (N = 846)	P-value
**Mother-related characteristics**								
Maternal Age	33 [30,35]	33 [30,35]	33 [30,36]	0.175	33 [30,35]	33 [31,36]	33 [30,36]	0.446
Multiple pregnancy	636 (34.0%)	1,368 (37.7%)	692 (34.8%)	0.011^*^	280 (33.1%)	285 (33.7%)	282 (33.3%)	0.967
Cesarean section	1,485 (79.3%)	2,726 (75.1%)	1,629 (81.9%)	<0.001^*,†^	690 (81.6%)	687 (81.2%)	685 (81.0%)	0.952
*In vitro* fertilization	408 (21.8%)	922 (25.4%)	405 (20.4%)	<0.001^*,†^	204 (24.1%)	182 (21.5%)	153 (18.1%)	0.010^**^
Gestational diabetes	142 (7.6%)	294 (8.1%)	140 (7.0%)	0.356	63 (7.4%)	80 (9.5%)	68 (8.0%)	0.306
Hypertensive disorder of pregnancy	461 (24.6%)	745 (20.5%)*	384 (19.3%)	<0.001^*,**^	173 (20.4%)	182 (21.5%)*	190 (22.5%)	0.602
Histologic chorioamnionitis	526 (30.5%)	1,083 (35.9%)	499 (33.2%)	0.001^*^	276 (32.6%)	287 (33.9%)	287 (33.9%)	0.807
Oligohydramnios	249 (13.9%)	539 (16.7%)	208 (11.4%)	<0.001^*,†^	110 (13.0%)	100 (11.8%)	115 (13.6%)	0.539
**Neonate-related characteristics**								
Gestational age at birth (weeks)	29^+4^ [27^+3^, 31^+3^]	28^+6^ [26^+4^, 30^+5^]	28^+4^ [26^+4^, 30^+4^]	<0.001^*,**^	28^+6^ [27^+0^, 30^+4^]	28^+6^ [27^+0^, 30^+3^]	28^+5^ [27^+1^, 30^+3^]	0.896
Birthweight (g)	1,190 [950, 1,370]	1,120 [850, 1.320]	1,120 [870, 1,320]	<0.001^*,**^	1,150 [920, 1,340]	1,120 [898, 1.310]	1,120 [890, 1,320]	0.262
Male gender	941 (50.2%)	1,806 (49.7%)	999 (50.2%)	0.912	437 (51.7%)	417 (49.3%)	428 (50.6%)	0.622
Small for gestational age	553 (29.5%)	941 (25.9%)	439 (22.1%)	<0.001^*,**,†^	174 (20.6%)	171 (20.2%)	176 (20.8%)	0.955
Steroid, complete	832 (59.3%)	1,733 (59.9%)	747 (51.6%)	<0.001^**,†^	489 (57.8%)	467 (55.2%)	457 (54.0%)	0.277
Delivery room resuscitation	70 (3.8%)	162 (4.5%)	91 (4.6%)	0.351	34 (4.0%)	26 (3.1%)	31 (3.7%)	0.572
Apgar score at 1 min	5 [4,6]	5 [3,6]	5 [3,6]	<0.001^*,**^	5 [4,6]	5 [3,7]	5 [3,6]	0.099
Apgar score at 5 min	7 [6,8]	7 [6,8]	7 [6,8]	<0.001^*,**^	7 [6,8]	7 [6,8]	7 [6,8]	0.264

Propensity score matching via gestational age, gender, small for gestational age, multiple pregnancies, cesarean section, hypertensive disorders of pregnancy, histologic chorioamnionitis, oligohydramnios, complete course of antenatal corticosteroid use, delivery room resuscitation, and 5 minute Apgar score.

Kruskal-Wallis test or chi-square test in both propensity score unmatched population and propensity score matched population.

*P < 0.05 between low sepsis group and intermediate sepsis group.

**P < 0.05 between low sepsis group and high sepsis group.

^†^P < 0.05 between intermediate sepsis group and high sepsis group.

**Table 3:** Distribution of groups according to the mean annual number of very-low-birth-weight infants registered in Korean Neonatal Network among three groups.

Groups	Low sepsis group (N = 1,873)	Intermediate sepsis group (N = 3,631)	High sepsis group (N = 1,989)
1	8 (0.4%)	124 (3.4%)	6 (0.3%)
2	118 (6.3%)	384 (10.6%)	138 (6.9%)
3	285 (15.2%)	579 (15.9%)	156 (7.8%)
4	200 (10.7%)	264 (7.3%)	426 (21.4%)
5	158 (8.4%)	384 (10.6%)	401 (20.2%)
6	716 (38.2%)	635 (17.5%)	406 (20.4%)
7	388 (20.7%)	1261 (34.7%)	456 (22.9%)
Median group (25^th^, 75^th^ percentile)	Group 6 (Group 4, Group 6)	Group 6 (Group 3, Group 7)	Group 5 (Group 4, Group 6)

Group 1; mean annual very-low-birthweight infants 〈10 patients per year, Group 2; ≥10 and <20, Group 3; ≥20 and <30, Group 4; ≥30 and <40, Group 5; ≥40 and <50, Group 6; ≥50 and <100, Group 7; ≥100.

P-value = 0.051 by Kruskal-Wallis test.

#### Neonatal outcomes of the three groups

Respiratory distress syndrome (RDS), patent ductus arteriosus (PDA) with treatment, periventricular leukomalacia (PVL), and moderate to severe bronchopulmonary dysplasia (BPD) were more frequent in the HS group when compared with the LS and IS groups. There was a serial increase in the rate of multiple courses of surfactant use, hypotension within one week after birth, necrotizing enterocolitis (NEC) ≥ stage 2, intraventricular hemorrhage (IVH) ≥ grade 3, severe BPD, BPD with postnatal steroid use, pulmonary hypertension requiring treatment, and mortality from the LS and IS to the HS group (Table 4). There were no significant differences in the duration of hospitalization between the three groups; however, it was shorter in the LS group when compared with the IS and HS groups in the survivors. There was also a serial increase in the duration of respiratory care, invasive ventilator care, and days to full feeding both in the total neonates and in the survivors from the LS and IS to the HS group (Table 4).

**Table 4:** Neonatal outcomes in the study population.

	Propensity score matching							
	Unmatched data set (N = 7,493)				Matched data set (N = 2,538)			
	Low sepsis group (N = 1,873)	Intermediate sepsis group (N = 3,631)	High sepsis group (N = 1,989)	P-value	Low sepsis group (N = 846)	Intermediate sepsis group (N = 846)	High sepsis group (N = 846)	P-value
Respiratory distress syndrome	1,396 (74.0%)	2,689 (74.1%)	1,728 (86.9%)	<0.001^**,†^	675 (79.8%)	623 (73.6%)	743 (87.8%)	<0.001^*,**,†^
Surfactant use	1,382 (73.8%)	2,781 (76.6%)	1,713 (86.1%)	<0.001^**,†^	682 (80.6%)	651 (77.0%)	739 (87.4%)	<0.001^**,†^
PDA with treatment	622 (41.2%)	1,215 (42.1%)	813 (50.8%)	<0.001^**,†^	306 (46.6%)	297 (44.3%)	341 (51.0%)	0.045^‡^
Hypotension within one week	299 (16.0%)	786 (21.6%)	620 (31.2%)	<0.001^*,**,†^	140 (16.5%)	174 (20.6%)	250 (29.6%)	<0.001^**,†^
NEC ≥ stage 2	69 (3.7%)	222 (6.1%)	163 (8.2%)	<0.001^*,**,†^	33 (3.9%)	43 (5.1%)	64 (7.6%)	0.003^**^
Isolated intestinal perforation	12 (0.6%)	71 (2.0%)	50 (2.5%)	<0.001^*,**^	5 (0.6%)	14 (1.7%)	22 (2.6%)	0.005^**^
IVH ≥ grade 3	92 (5.0%)	314 (8.9%)	213 (11.0%)	<0.001^*,**,†^	40 (4.8%)	61 (7.4%)	80 (9.7%)	0.001^**^
PVL	105 (5.7%)	244 (7.0%)	181 (9.4%)	<0.001^**,†^	55 (6.6%)	55 (6.7%)	84 (10.2%)	0.008^**,†^
Moderate to severe BPD	385 (22.4%)	811 (25.3%)	580 (34.8%)	<0.001^**,†^	193 (24.6%)	197 (25.7%)	255 (34.9%)	<0.001^**,†^
Severe BPD	186 (10.8%)	486 (15.2%)	355 (21.3%)	<0.001^*,**,†^	94 (12.0%)	104 (13.6%)	155 (21.2%)	<0.001^**,†^
BPD with steroid use	206 (11.0%)	639 (17.6%)	514 (25.8%)	<0.001^*,**,†^	86 (10.2%)	140 (16.5%)	214 (25.3%)	<0.001^*,**,†^
Pulmonary hypertension requiring treatment	75 (4.0%)	227 (6.3%)	187 (9.4%)	<0.001^*,**,†^	47 (5.6%)	54 (6.4%)	77 (9.1%)	0.012^**^
ROP needs surgery or VEGF	143 (12.7%)	336 (14.5%)	167 (14.6%)	0.342	83 (16.8%)	76 (14.3%)	76 (16.4%)	0.500
Death	152 (8.1%)	451 (12.4%)	344 (17.3%)	<0.001^*,**,†^	67 (7.9%)	87 (10.3%)	123 (14.5%)	<0.001^**,†^
Discharge with respiratory support	261 (15.2%)	562 (17.7%)	319 (19.4%)	0.005^**^	129 (16.6%)	136 (17.9%)	152 (21.0%)	0.075
Hospital day	56 [40,79]	58 [38,84]	57 [37,79]	0.067	61 [44,83]	59 [41,82]	58 [41,79]	0.031^**^
Duration of invasive ventilator care	2 [0, 8]	3 [0, 17]	4 [1,19]	<0.001^*,**,†^	2 [0, 9]	3 [0, 15]	4 [1,18]	<0.001^**,†^
Duration of total respiratory support	19 [5,46]	25 [6,55]	31 [10,58]	<0.001^*,**,†^	25 [7,51]	28 [7,55]	32 [11,57]	<0.001^**,†^
Days to full feeding	13 [8,21]	15 [8,29]	19 [10,35]	<0.001^*,**,†^	14 [9,24]	15 [8,29]	19 [10,35]	<0.001^**,†^
**In survivors**								
Hospital day	58 [43,80]	62 [45,88]	62 [46,84]	<0.001^*,**^	64 [47,84]	62 [45,84]	62 [47,82]	0.429
Duration of invasive ventilator care	2 [0, 7]	2 [0, 14]	3 [1,17]	<0.001^*,**,†^	2 [0, 8]	3 [0, 12]	3 [1,17]	<0.001^**,†^
Duration of total respiratory support	21 [5,47]	28 [6,57]	35 [12,61]	<0.001^*,**,†^	27 [7,53]	30 [8,56]	35 [12,59]	<0.001^**,†^
Days to full feeding	12 [8,21]	15 [8,29]	18 [10,34]	<0.001^*,**,†^	14 [9,24]	15 [8,29]	19 [10,35]	<0.001^**,†^

Propensity score matching via gestational age, gender, small for gestational age, multiple pregnancies, cesarean section, hypertensive disorders of pregnancy, histologic chorioamnionitis, oligohydramnios, complete course of antenatal corticosteroid use, delivery room resuscitation, and 5 minute Apgar score.

Kruskal-Wallis test or chi-square test in both propensity score unmatched population and propensity score matched population.

*P < 0.05 between low sepsis group and intermediate sepsis group.

**P < 0.05 between low sepsis group and high sepsis group.

^†^P < 0.05 between intermediate sepsis group and high sepsis group.

‡Not statistically significant after Bonferroni correction.

PDA; patent ductus arteriosus, BPD; bronchopulmonary dysplasia, IVH; intraventricular hemorrhage, PVL; periventricular leukomalacia, NEC; necrotizing enterocolitis, ROP; retinopathy of prematurity, VEGF; vasculoendothelial growth factor.

### Differences between the three groups after the propensity score matching

#### Maternal demographics and neonatal characteristics of the three groups

After the propensity score matching, there was no significant difference in the maternal and neonatal demographic characteristics between the three groups. Only the rate of *in-vitro* fertilization was higher in the LS group when compared with the HS group (Table 2).

#### Neonatal outcomes

After the propensity score matching, surfactant use, hypotension within one week, PVL, moderate to severe BPD and severe BPD were more frequent in the HS group when compared with the LS and IS groups. Mortality was also higher in the HS group when compared with the LS or IS group. The length of stay in hospitals for the survivors was not different between the three groups; however, the duration of invasive ventilator care and the duration of total respiratory care were longer in the HS group when compared with the LS and IS groups in the survivors. Days to full feeding were also longer in the HS group (Table 4).

Both univariate logistic regression analysis after the propensity score matching and multivariate logistic regression analysis with random intercepts adjusting for GA and the hospital groups according to the mean annual number of VLBW infants registered in the KNN after the propensity score matching showed an increased odds ratio (OR, >1) for mortality, moderate to severe BPD or death, and PVL in HS group when compared with IS group and with LS group. The OR of survival without major morbidities in the HS group was 0.7 when compared with the IS group and 0.7 with LS group. However, those outcomes were not much different when compared between the IS and LS groups (Table 5).

**Table 5:** Logistic regression analysis with random effect after propensity score matching.

	Univariate logistic regression			Multivariate logistic regression adjusted for gestational age and groups according to the mean annual number of very-low-birth-weight infants		
	IS group versus LS group	HS group versus IS group	HS group versus LS group	IS group versus LS group	HS group versus IS group	HS group versus LS group
Death	1.3 (0.95–1.86)	1.5 (1.11–1.99)	2.0 (1.44–2.71)	1.1 (0.79–1.66)	2.0 (1.39–2.76)	2.2 (1.57–3.19)
Moderate to severe BPD	1.1 (0.90–1.45)	1.5 (1.20–1.91)	1.7 (1.37–2.18)	1.2 (0.90–1.51)	1.7 (1.34–2.24)	2.0 (1.57–2.61)
Severe BPD	1.2 (0.89–1.63)	1.7 (1.29–2.27)	2.1 (1.55–2.75)	1.2 (0.87–1.66)	2.0 (1.45–2.69)	2.4 (1.74–3.24)
Moderate to severe BPD or death	1.2 (0.96–1.47)	1.6 (1.30–1.96)	1.9 (1.54–2.34)	1.2 (0.93–1.51)	1.9 (1.50–2.42)	2.3 (1.78–2.87)
Severe BPD or death	1.2 (0.98–1.59)	1.7 (1.36–2.14)	2.1 (1.69–2.68)	1.2 (0.92–1.59)	2.1 (1.65–2.80)	2.6 (1.99–3.40)
PVL	1.2 (0.84–1.86)	1.6 (1.07–2.27)	1.9 (1.34–2.82)	1.3 (0.85–1.89)	1.5 (1.06–2.26)	2.0 (1.34–2.86)
Survival without major morbidities*	1.1 (0.89–1.34)	0.7 (0.54–0.83)	0.7 (0.59–0.91)	1.1 (0.90–1.38)	0.7 (0.53–0.83)	0.7 (0.59–0.92)

*Major morbidities include moderate to severe BPD, NEC ≥ stage 2, IVH ≥ grade 3, PVL, ROP requiring surgery or VEGF.

HS; high sepsis, IS; intermediate sepsis, LS; low sepsis, BPD; bronchopulmonary dysplasia, PVL; periventricular leukomalacia.

### Differences in the distribution of pathogens between the three groups

There were significant differences in the distribution of pathogens between the three groups both in the propensity score unmatched group (P = 0.001) and in the propensity score matched group (P < 0.001). The proportion of sepsis by gram positive organisms was higher, and the proportion of sepsis by gram negative organisms was lower in the HS group when compared with the IS and LS groups. After the propensity score matching, the rate of gram-negative sepsis was lower in the HS group when compared with the LS group. There was no significant difference in the rate of fungal sepsis between the three groups (Table 6).

**Table 6:** Distribution of Pathogens among three groups both in unmatched and matched dataset.

	Propensity score matching							
	Unmatched dataset (N = 7,493)				Matched dataset (N = 2,538)			
	Low sepsis group	Intermediate sepsis group	High sepsis group	P-value	Low sepsis group	Intermediate sepsis group	High sepsis group	P-value
Gram positive organisms	90 (60.8%)	535 (68.9%)	767 (78.6%)	<0.001^**,†^	42 (56.8%)	128 (76.6%)	328 (79.6%)	<0.001^*,**^
Staphylococcus coagulase negative	35 (23.6%)	253 (32.6%)	428 (43.9%)		17 (23.0%)	50 (29.9%)	174 (42.2%)	
Staphylococcus aureus coagulase positive	18 (12.2%)	106 (13.6%)	104 (10.7%)		6 (7.6%)	37 (21.8%)	53 (12.6%)	
Staphylococcus other	17 (11.5%)	97 (12.5%)	117 (12.0%)		7 (9.5%)	22 (13.2%)	61 (14.8%)	
Streptococcus other	5 (3.4%)	17 (2.2%)	11 (1.1%)		4 (5.4%)	4 (2.4%)	1 (0.2%)	
Streptococcus group B	8 (5.4%)	12 (1.5%)	9 (0.9%)		5 (6.8%)	3 (1.8%)	2 (0.5%)	
Enterococcus species	7 (4.7%)	49 (6.3%)	98 (10.0%)		3 (4.1%)	12 (7.2%)	38 (9.2%)	
Listeria species	0 (0.0%)	1 (0.1%)	0 (0.0%)		0 (0.0%)	0 (0.0%)	0 (0.0%)	
Gram negative organisms	37 (25.0%)	163 (21.0%)	125 (12.8%)	<0.001^**,†^	22 (29.7%)	28 (16.8%)	57 (13.8%)	0.003^**^
Escherichia coli	13 (8.8%)	37 (4.8%)	21 (2.2%)		6 (8.1%)	10 (6.0%)	12 (2.9%)	
Klebsiella species	10 (6.8%)	34 (4.4%)	23 (2.4%)		7 (9.5%)	4 (2.4%)	12 (2.9%)	
Pseudomonas species	3 (2.0%)	8 (1.0%)	14 (1.4%)		1 (1.4%)	2 (1.2%)	5 (1.2%)	
Acinetobacter species	4 (2.7%)	20 (2.6%)	9 (0.9%)		3 (4.1%)	3 (1.8%)	4 (1.0%)	
Enterobacter species	4 (2.7%)	33 (4.2%)	28 (2.9%)		3 (4.1%)	6 (3.6%)	13 (3.2%)	
Burkholderia species	0 (0.0%)	16 (2.1%)	3 (0.3%)		0 (0.0%)	2 (1.2%)	1 (0.2%)	
Clostridium species	0 (0.0%)	1 (0.1%)	0 (0.0%)		0 (0.0%)	0 (0.0%)	0 (0.0%)	
Stenotrophomonas maltophilia	1 (0.7%)	3 (0.4%)	4 (0.4%)		1 (1.4%)	0 (0.0%)	1 (0.2%)	
Achromobacter species	1 (0.7%)	0 (0.0%)	0 (0.0%)		1 (1.4%)	0 (0.0%)	0 (0.0%)	
Citrobacter species	0 (0.0%)	1 (0.1%)	0 (0.0%)		0 (0.0%)	0 (0.0%)	0 (0.0%)	
Hemophilus species	0 (0.0%)	1 (0.1%)	1 (0.1%)		0 (0.0%)	0 (0.0%)	0 (0.0%)	
Moraxella species	0 (0.0%)	0 (0.0%)	1 (0.1%)		0 (0.0%)	0 (0.0%)	0 (0.0%)	
Proteus species	0 (0.0%)	1 (0.1%)	0 (0.0%)		0 (0.0%)	0 (0.0%)	0 (0.0%)	
Serratia species	1 (0.7%)	8 (1.0%)	16 (1.6%)		0 (0.0%)	1 (0.6%)	6 (1.5%)	
Bacteroides species	0 (0.0%)	0 (0.0%)	5 (0.5%)		0 (0.0%)	0 (0.0%)	3 (0.7%)	
Fungus	12 (8.1%)	65 (8.4%)	71 (7.3%)	0.691	5 (6.8%)	8 (4.8%)	23 (5.6%)	0.788
Candida	10 (6.8%)	63 (8.1%)	65 (6.7%)		5 (6.8%)	8 (4.8%)	21 (5.1%)	
Yeast, others	2 (1.4%)	2 (0.3%)	6 (0.6%)		0 (0.0%)	0 (0.0%)	2 (0.5%)	
Others	9 (6.1%)	14 (1.8%)	13 (1.3%)	0.002^*,**^	5 (6.8%)	3 (1.8%)	4 (1.0%)	0.007^**^
Total	148 (100.0%)	777 (100.0%)	976 (100.0%)		74 (100.0%)	167 (100.0%)	412 (100.0%)	

Organisms counts differ from the infants with sepsis due to the multiple episode of sepsis. *P < 0.05 between low sepsis group and intermediate sepsis group. **P < 0.05 between low sepsis group and high sepsis group. ^†^P < 0.05 between intermediate sepsis group and high sepsis group.

### Differences between the three groups when we excluded the infants with EOS

The sepsis incidence density and the sepsis rate were serially increased from the LS and IS to the HS group both before and after propensity score matching (Supplement Table 1). When we performed the same analyses in infants without EOS, there were significant differences in GA, birthweight, multiple pregnancy, cesarean section, hypertensive disorder of pregnancy, SGA, HCA, oligohydramnios, complete course of ACS use, and 1 minute and 5 minute Apgar scores. After propensity score matching was done, there were no significant differences between the LS, IS, and HS groups in maternal demographics and neonatal characteristics except 1 minute Apgar score (Supplement Table 2).

The incidences of RDS, hypotension within one week, PVL, moderate to severe BPD, and severe BPD were significantly higher in HS group when compared with IS and LS groups both before and after the propensity score matching (Supplement Table 3). Both univariate logistic regression analysis after the propensity score matching and multivariate logistic regression analysis with random intercepts adjusting for GA and the hospital groups according to the mean annual number of VLBW infants registered in the KNN after the propensity score matching showed an increased OR (>1) for mortality, moderate to severe BPD and/or death, and PVL in HS group when compared with IS group and with LS group. Also, the OR of survival without major morbidities in the HS group was significantly decreased (<1) in the HS group when compared with the IS and LS groups both before and after the propensity score matching (Supplement Table 4).

## Discussion

In this study using nationwide database, we demonstrated that the incidence of sepsis showed considerable center-to-center variability in Korea. This difference remained significant even after the propensity score matching for multiple antenatal and perinatal factors including GA and gender. In our study, the sepsis rate serially increased from the LS, IS to the HS group. The rate of LOS was also increased from the LS group to the HS group, and especially gram-positive sepsis was more frequent in the HS group (79%) when compared with the LS group (61%). The maternal demographics and neonatal general characteristics showed that the GA and birthweight were higher in the LS group when compared with the IS and HS groups; however, there was no significant difference in GA and birthweight between the IS group and the HS group. After the propensity score matching for such different general characteristics, the neonatal outcomes were worse in the HS group when compared with the LS and IS groups; however, there was no significant difference in neonatal outcomes between the LS and IS groups. When we performed the same analyses in infants without EOS, the results were also same.

A study by the National Institute of Child Health and Human Development Neonatal Research Network (NICHD) on 6,215 infants who survived beyond 3 days showed that the rate of LOS for at least one episode was 21%, and the rate for the first episode of LOS was 3.1 per 1,000 patient days, which are similar to our results^1^. In the Canadian Neonatal Network, 15% of infants with less than 32 weeks of gestation suffered from LOS, and bacterial late-onset sepsis was associated with mortality and BPD^3^. They concluded that the risk factors for LOS were a lower gestation, higher Score for Neonatal Acute Physiology (version II scores), the presence of prolonged use of central catheters, parenteral nutrition, and nothing by mouth^3^. In our study, similar results were seen in that the GA in the LS group was significantly higher when compared to the IS or HS group. In the LS group, days to full feeding were shorter with 14 postnatal days when compared to the HS group with 19 postnatal days until full feeding.

In our study, a group of infants with a higher sepsis rate had poorer neonatal outcomes even when there were no significant differences in GA and birthweight between the IS and HS groups. Because we did not assess whether sepsis itself is an independent risk factor of mortality, BPD, and PVL in this study, we still cannot determine whether sepsis itself was the direct leading cause of the poorer neonatal outcomes or other factors that were associated with a higher sepsis rate led to such poorer neonatal outcomes. From many previous reports, numerous evidence has shown that sepsis itself is associated with increased mortality, BPD, and long-term neurodevelopmental outcomes in preterm infants^1–5^. Additionally, the many nationwide quality improvements by nationwide neonatal networks, such as Vermont-Oxford Network^6^ and Canadian Neonatal Network^14^, to reduce sepsis in units with a high sepsis rate, especially focusing on the LOS, were the most effective in improving neonatal outcomes^6,14^. Such a reduction in LOS also improved the survival of extremely preterm infants born at 23–26 weeks of gestation in a single tertiary hospital^15^. Moreover, quality improvement can have a spill-over effect from one outcome to another, and many common strategies are used to improve different outcomes. For example, the nosocomial infection rate was decreased not only in the evidence-based practice from improving quality methods (EPIQ) infection group but also in the EPIQ pulmonary group in the EPIQ trial by the Canadian Neonatal Network, which implies that decreasing the duration of invasive ventilator care is another important method to reduce the sepsis rate^14^. Nowadays, the Canadian Neonatal Network and Vermont-Oxford Network have adopted a multi-targeting quality improvement campaign simultaneously to improve the composite of morbidity and major neonatal outcomes, especially in extremely low GA infants^16,17^. That is why we think that nationwide quality improvement to control sepsis should be started first in Korea, considering that we cannot start multiple strategies for quality improvement at once.

In our study, in only the HS group, the mortality and BPD were increased. Actually, the gap in the incidence density of sepsis is larger between the HS and IS groups when compared with the gap between the IS and LS groups in our study population. Thus, decreasing the sepsis rate in the HS group will be very important and will be a main target of quality improvement in NICUs. Among the LOS infants, coagulase-negative staphylococcus (CONS) is known to be the most prevalent cause of sepsis^12^; however, gram-negative or fungal sepsis is known to be associated with the highest risk of mortality or adverse outcomes in preterm infants^3,18^. CONS is also a well-known bacteria associated with an increased risk of BPD^19,20^. Gram-positive organisms are usually associated with catheter-related bloodstream infection, and gram-negative organisms are more commonly seen in NEC patients^18^. In our cohort, CONS was the most prevalent causal organisms of sepsis, especially in the HS group, and there was no significant difference in the prevalence of fungal sepsis between the three groups. A quality improvement program with strategies to reduce CONS sepsis such as a central catheter bundle, reducing the duration of central catheter insertion, and hand washing will be important in improving neonatal outcomes in the NICUs of South Korea. Because the duration of invasive ventilator care and the days to full feeding were longer in the HS group, reducing invasive ventilation, prompt extubation as soon as possible, and an earlier start of enteral feeding will be also helpful to reduce the sepsis rate and to improve neonatal outcomes.

Many previous reports showed that there are variations in practice and in neonatal outcomes between NICUs^8^. Moreover, there was a significant disparity in the sepsis incidence between units, which is common in other countries^9–11^. In the NICHD cohort, there was center-to-center variability in the incidence of LOS ranging from 10.6% to 31.7%^1^. In our study population, the median sepsis rate was 6.7% in the LS group; however, it approached 36.2% in the HS group. Actually, there was no significant difference in the maternal demographics and neonatal baseline characteristics between the IS and HS groups in our study. However, the neonatal outcome was worse in the HS group when compared with the IS group, which implies that other factors besides GA and birth weight might influence the neonatal outcomes in each NICU. The Canadian Neonatal Network searched for the risk factors for the variations in the rates of nosocomial infections among each unit, and after adjusting for risk factors associated with nosocomial infections such as GA, birth weight, and disease severity, there was still a difference in the rate of nosocomial infections between units^11^. However, there was little doubt that the nosocomial infections were associated with significant in-hospital morbidities in that study^11^. In our study, only in the HS group, adverse neonatal morbidities were more frequent.

To reduce the gap in the sepsis incidence rate between units, assessing which factors were different in relation to the occurrence of sepsis and searching for the factors associated with sepsis in the units with the lowest and highest infection rates would be helpful to identify the medical or nursing care practices influencing the sepsis rate. According to previous reports, number of days of central catheter insertion and total parenteral nutrition, resuscitation at birth, status of infants on admission, patient load or nurse to patient ratio were very important risk factors of sepsis^3,9,10^. Besides well-known risk factors of sepsis such as GA and patient status at admission, the differences in daily clinical practices caused the center-to-center variation of the sepsis rate^11^. Unfortunately, in this study, we could not get such information from the KNN database; we could only get the information about the group of mean annual number of VLBW infants registered in KNN, which showed no significant difference between three groups. When we performed the multivariate logistic regression analysis with random intercepts adjusting for GA and the hospital groups after the propensity score matching, the results were not much changed from those of the analysis with multivariate logistic regression only adjusting for GA after propensity score matching. Those groups can represent some portion or quality of the NICU size; however, more information about the NICU levels, i,e, secondary, tertiary, or quarternary units, the presence of other supporting parts such as neonatal surgery, and the presence of a perinatal center or regional NICU center are needed to be judged first whether the hospital level influences the sepsis rate. Thus, a further prospective large cohort study is needed to find such risk factors.

In contrast to other reports^21^, the length of stay in hospital was not different between the three groups after the propensity score matching in the survivors of our cohort. However, the duration of invasive ventilator care or respiratory support and days to full feeding were longer in the HS group, which were similar to the results in other reports^1^. It means that the disease severity of the infants in the HS group was higher than that in the LS and IS groups. Especially, between the IS and HS groups, there was no difference in the GA and birth weight; however, morbidities, duration of invasive ventilator care, and the duration of hospitalization were longer in the HS group. Additionally, a longer duration of ventilator care and longer days to full feeding, which could suggest a longer duration of central catheter insertion, are well-known risk factors of sepsis.

The limitations of our study were as follows; first, we could not perform a cluster analysis at the individual hospital level at which the individual infant was admitted because it was a policy that the KNN did not provide any data about the individual hospital at which any infant was admitted. We tried to compensate for those problems by performing the multivariate logistic regression analysis with random intercepts adjusting for the hospital groups divided according to the number of registered VLBWs in the KNN. Second, we still could not clearly determine whether sepsis itself or other factors associated with a higher sepsis rate led to such poorer neonatal outcomes. Further studies should be done focusing on this problem. Third, we could not collect information about the duration of central catheter use although days to full feeding can be an indicator suggesting the duration of central catheter use. We also could not get information on staff such as the nurse to patient ratio and the unit size such as tertiary or secondary hospital. Further studies will be needed to determine the risk factors that lead to the difference in the incidence of sepsis between the three groups including the staff and facilities of the NICUs. Fourth, we could not determine the order of the outcomes, so we could not determine the cause and result relationships between sepsis and other outcomes except for BPD or ROP. That’ is why we performed logistic regression analysis for only death, BPD, PVL, and survival without major morbidities. Fifth, we could not get information on the duration of antibiotic use and the antibiotic resistances of each organism from our KNN database. Considering that antibiotic resistance can also be a risk factor for a longer duration of antibiotic use and disease severity, a more sophisticated design for a prospective study including such information should be done later.

However, the strength of our study is that we used a nationwide cohort with a large sample size including almost all the neonates born from NICUs in South Korea. Our study cohort was also achieved by standardized data collection, which improved the accuracy of the data analysis. Moreover, this study is the first report to demonstrate the differences in neonatal outcomes according to the different units with variable sepsis rates in South Korea.

## Conclusion

The sepsis rate was markedly different from the LS group to the HS group ranging from 6.7% to 36.2%. The gap in the incidence of sepsis between the IS and HS groups was higher than the gap between the LS and IS groups. The GA and birthweight were more mature and heavier in the LS group when compared with the IS or HS group; however, there was no difference in the GA and birthweight between the IS and HS groups. Adverse neonatal outcomes including mortality, BPD, and PVL were higher in the HS group when compared with the LS and IS groups; however, there were no significant differences between the LS and IS groups, even after the propensity score matching. Other factors besides GA and birthweight in the HS group might be associated with worse neonatal outcomes. A future prospective cohort study that includes antibiotic use, patient care techniques, hand washing rate, and catheter insertion duration to assess the risk factors associated with center to center variability will be helpful to perform further nationwide interventions for quality improvement in infection control at NICUs.

## Methods

### Ethics statement

The KNN registry was approved by the institutional review board of each participating hospital including the Seoul Metropolitan Government Seoul National University (SMG-SNU) Boramae Medical Center (IRB number 26-2014-12). The names of all the participating hospitals were listed in the acknowledgement section. Informed consent was obtained from the parents and/or legal guardians of all the study participants at enrollment. All data collection was performed prospectively according to the guidelines and regulations of the KNN. All the researchers in each hospital put their data on digitally recording online case record forms, and such data are monitored regularly by KNN data management committee. The screening failure rate of KNN registry was 3.7% in 2016. The present study was approved by the KNN data management committee, and the IRB approval was exempted by the SMG-SNU Boramae Medical Center (IRB number 07-2018-4).

### Study population

This was a population-based observational study of VLBW infants born between 2013 and 2016, registered in the KNN database. The exclusion criteria were infants transferred to a ward, pediatric intensive care unit or other hospital, infants with major congenital anomalies, infants with no data about sepsis, and infants admitted for more than one year. Then, infants were classified into the LS, IS, or HS group according to the sepsis rate at each hospital. Propensity score matching was done to compensate for the differences in the maternal and neonatal demographic characteristics.

### Data collection and analysis

The data for the infants were generated and analyzed from the KNN database. Mother-related variables were compared such as maternal age, multiple pregnancies, *in vitro* fertilization, delivery mode, gestational diabetes, hypertensive disorders of pregnancy, HCA, and oligohydramnios. Neonate-related variables were also compared including GA, birthweight, gender, SGA, complete course of ACS use, delivery room resuscitation, and 1- and 5-minute Apgar scores. We also compared the distribution of groups according to the mean annual number of VLBW infants registered in KNN database. The following outcomes were collected for the analysis: mortality, RDS, PDA with treatment, BPD, IVH ≥ grade 3 for the Papile classification^22^, NEC ≥ stage 2 for the modified Bell’s criteria^23^, retinopathy of prematurity (ROP) requiring surgery or vascular endothelial growth factor (VEGF) treatment, discharge with respiratory support, the duration of hospitalization, and the duration of ventilator care and total respiratory support, and days to full feeding. And we compared the distribution of pathogens among LS, IS, and HS groups.

### Definitions

Sepsis was defined as the presence of clinical symptoms and signs with proven causative organisms documented from blood cultures, and the patients should receive at least five days of antibiotics as a sepsis treatment. If more than one organism was grown in the blood culture simultaneously, the decision about the causal organism is dependent on the opinion of the physician. If the organisms were identified from a specimen obtained within 3 postnatal days, it was defined as EOS. If the organisms were identified from a specimen obtained after 3 or more postnatal days, it was defined as LOS.

Infants were classified into the LS, IS, and HS group according to the percentile of sepsis incidence in each hospital. The LS, IS, and HS groups were defined as follows: when the sepsis incidence was less than 25 percentile, 25–74 percentile, and more than 75 percentile in the whole KNN registered hospital.

The sepsis (incidence) rate was calculated as the number of neonates who suffered from sepsis at least one time during hospitalization divided by the total number of neonates in each group. The incidence density of sepsis was calculated as the number of episodes of sepsis divided by the number of patient-days at risk.

We categorized our participating hospitals according to the mean annual number of VLBW infants registered in the KNN. The criteria were as following; group 1 < 10 (patients per year), 10 ≤ group 2 < 20, 20 ≤ group 3 < 30, 30 ≤ group 4 < 40, 40 ≤ group 5 < 50, 50 ≤ group 6 < 100, and group 7 ≥ 100.

SGA was defined as a birthweight less than the 10^th^ percentile based on the growth curve of Olsen *et al*.^24^ Hypertensive disorders of pregnancy were defined as any maternal diagnoses of chronic hypertension or pregnancy induced hypertension including preeclampsia, eclampsia, or hemolysis, elevated liver enzymes, and low platelet count syndrome. HCA was defined and classified using the grading by Salafia *et al*.^25^ Oligohydramnios was defined as amniotic fluid index <5. Delivery room resuscitation was defined as any cardiac compression or administration of medication in the delivery room. Complete course of ACS use was defined as the administration of 4 doses of dexamethasone or 2 doses of betamethasone, and the initial dose was given more than 24 hours and less than 7 days before birth. RDS was defined as chest radiographic findings consistent with RDS such as a diffuse ground glass appearance together with an oxygen requirement of more than 0.4 fractions of inspired oxygen^26^. Surfactant use was defined as the administration of any surfactant use regardless of purpose. PDA with treatment was defined as any pharmacologic or surgical treatment about preterm PDA. BPD was defined and classified according to the definition by Jobe AH *et al*.^27^. Pulmonary hypertension requiring treatment was defined as any inhaled nitric oxide, sildenafil, iloprost, bosentan, milrinone treatment for pulmonary hypertension. We defined the duration of invasive ventilator care as the duration of ventilator support by endotracheal tube. We also defined the duration of respiratory support as the whole duration of any invasive/non-invasive ventilator care or oxygen support during hospitalization.

### Statistical analysis

All the continuous variables were expressed as the median and interquartile range (IQR), and the categorical variables were expressed as the numbers and proportions. Comparisons of the maternal demographics and neonatal characteristics among the LS, IS, and HS groups were performed using one-way analysis of variance (ANOVA) test for continuous variables and chi-square test for categorical variables. Comparison of the groups according to the mean annual number of VLBW infants registered in KNN was performed using Kruskal-Wallis test. For multiple comparisons, Bonferroni correction was applied. Because the GA and birthweight were significantly higher in the LS group when compared with the IS or HS group and there were significant differences in the many maternal and neonatal demographics among the three groups, propensity score matching was considered to minimize the selection bias. To derive the propensity scores, multivariate logistic regression for the pairwise groups was conducted with GA, gender, SGA, multiple pregnancies, cesarean section, hypertensive disorders of pregnancy, HCA, oligohydramnios, complete course of steroid use, delivery room resuscitation, and 5-minute Apgar score. Since the correlation between GA and birthweight was highly positive, only GA was included as an adjusting factor in the propensity score matching. By first matching the LS to IS, LS to HS, and the IS and HS groups based on their propensity scores using the nearest neighbor matching algorithm, three-group propensity score matched dataset was generated. Univariate and multivariate logistic regression analyses with random intercepts were performed to account for hospital group effects according to the mean annual number of VLBWs registered in KNN, with adjustment for GA on mortality, moderate to severe BPD, severe BPD, PVL, and survival without major morbidities (moderate to severe BPD, NEC ≥ stage 2, IVH ≥ grade 3, PVL, ROP requiring surgery or VEGF treatment) in matched sets. Comparison of the distribution of pathogens between three groups were performed by chi-square test. All analyses were performed with R version 3.5.0 (http://www.r-project.org) with the statistical significance set at a *P*-value <0.05.

## Supplementary information

Supplementary information.

## Data Availability

The dataset analyzed in this study are not publicly available due to the policy of Research of Korea Centers for Disease Control and Prevention. However, dataset are available from the corresponding author on reasonable request.
