# Supplementary material for: The Influence of the Variation in Sepsis Rate between Neonatal Intensive Care Units on Neonatal Outcomes in Very-Low-Birth-Weight Infants
Source: Sci Rep. 2020 Apr 21;10:6687. doi: 10.1038/s41598-020-63762-6 (PMC7174287; doi:10.1038/s41598-020-63762-6)
Supplement: Supplementary file 1 — Supplementary information. [file 41598_2020_63762_MOESM1_ESM.docx]

**Supplementary Informations**

**Title:** **The Influence of the Variation in Sepsis Rate between Neonatal Intensive Care Units on Neonatal Outcomes in Very-Low-Birth-Weight Infants**

**Authors**

Tae-Jung Sung^1^, Jin A Sohn^2,3^, Sohee Oh^4^, Jin A Lee^2,3^*

**Affiliations**

*Department of Pediatrics, Hallym University Medical Center kangnam Sacred Heart Hospital, Seoul, South Korea^1^*

*Department of Pediatrics, Seoul National University College of Medicine, Seoul, South Korea^2^*

*Department of Pediatrics, SMG-SNU Boramae Medical Center, Seoul, South Korea^3^*

*Department of Biostatistics, SMG-SNU Boramae Medical Center, Seoul, South Korea^4^*

Supplement Table 1. General characteristics about sepsis in the study population excluding infants with early-onset sepsis

|  | Propensity score matching | | | | | | | |
| --- | --- | --- | --- | --- | --- | --- | --- | --- |
|  | Unmatched data set (N=7,363) | | | | Matched data set (N=2,130) | | | |
|  | Low sepsis group  (N=1,847) | Intermediate sepsis group  (N=3,579) | High sepsis group  (N=1,937) | *P*-value | Low sepsis group  (N=710) | Intermediate sepsis group  (N=710) | High sepsis group  (N=710) | *P*-value |
| Sepsis incidence density (cases per 1,000 person-day) | 0.93 | 2.90 | 8.24 | <0.001^*,**,†^ | 0.81 | 2.98 | 8.45 | <0.001^*,**,†^ |
| Sepsis, total,  number (%) | 99 (5.4%) | 563 (15.7%) | 669 (34.5%) | <0.001^*,**,†^ | 40 (5.6%) | 115 (16.2%) | 260 (36.6%) | <0.001^*,**,†^ |
| Sepsis, bacterial,  number (%) | 89 (4.8%) | 528 (14.8%) | 467 (33.4%) | <0.001^*,**,†^ | 36 (5.1%) | 108 (15.2%) | 255 (35.9%) | <0.001^*,**,†^ |
| Sepsis, Fungal,  number (%) | 10 (0.5%) | 62 (1.7%) | 61 (3.1%) | <0.001^*,**,†^ | 4 (0.6%) | 12 (1.7%) | 19 (2.7%) | 0.007^**^ |
| Sepsis, multiple,  number (%) | 10 (0.5%) | 104 (2.9%) | 157 (8.1%) | <0.001^*,**,†^ | 4 (0.6%) | 19 (2.7%) | 56 (7.9%) | <0.001^*,**,†^ |
| First postnatal day of sepsis(days) | 19 [10, 31] | 17 [9, 30] | 17 [10, 28] | 0.688 | 17 [9, 28] | 18 [10, 30] | 17 [11, 29] | 0.944 |

Propensity score matching via gestational age, gender, small for gestational age, multiple pregnancies, cesarean section, hypertensive disorders of pregnancy, histologic chorioamnionitis, oligohydramnios, complete course of antenatal corticosteroid use, delivery room resuscitation, and 5 minute Apgar score

Kruskal-Wallis test or chi-square test in both propensity score unmatched population and propensity score matched population

*P<0.05 between low sepsis group and intermediate sepsis group

** P<0.05 between low sepsis group and high sepsis group

†P<0.05 between intermediate sepsis group and high sepsis group

Supplement Table 2. Maternal demographics and neonatal characteristics in the study population without infants with early-onset sepsis

|  | Propensity score matching | | | | | | | |
| --- | --- | --- | --- | --- | --- | --- | --- | --- |
|  | Unmatched data set (N=7,363) | | | | Matched data set (N=2,130) | | | |
|  | Low sepsis group  (N=1,847) | Intermediate sepsis group  (N=3,579) | High sepsis group  (N=1,937) | P-value | Low sepsis group  (N=710) | Intermediate sepsis group  (N=710) | High sepsis group  (N=710) | P-value |
| **Mother-related characteristics** | | | | | | | | |
| Maternal Age | 33 [30, 35] | 33 [30, 35] | 33 [30, 36] | 0.163 | 33 [31, 35] | 33 [30, 36] | 33 [31, 36] | 0.932 |
| Multiple pregnancy | 631 (34.2%) | 1,354 (37.8%) | 677 (35.0%) | 0.013^*^ | 241 (33.9%) | 242 (34.1%) | 254 (35.8%) | 0.722 |
| Cesarean section | 1,471 (79.6%) | 2,697 (75.4%) | 1,587 (81.9%) | <0.001^*,†^ | 589 (83.0%) | 578 (81.4%) | 587 (82.7%) | 0.717 |
| In vitro fertilization | 403 (21.8%) | 909 (25.4%) | 396 (20.4%) | <0.001^*,†^ | 171 (24.1%) | 166 (23.4%) | 144 (20.3%) | 0.190 |
| Gestational diabetes | 140 (7.6%) | 288 (8.0%) | 138 (7.1%) | 0.461 | 47 (6.6%) | 67 (9.4%) | 57 (8.0%) | 0.148 |
| Hypertensive disorder of pregnancy | 459 (24.9%) | 732 (20.5%) | 375 (19.4%) | <0.001^*,**^ | 150 (21.1%) | 139 (19.6%) | 152 (21.4%) | 0.656 |
| Histologic chorioamnionitis | 509 (29.9%) | 1,061 (35.7%) | 475 (32.5%) | <0.001^*^ | 246 (34.7%) | 247 (34.8%) | 243 (34.2%) | 0.973 |
| Oligohydramnios | 246 (13.9%) | 532 (16.8%) | 204 (11.4%) | <0.001^*,†^ | 105 (14.8%) | 104 (14.7%) | 98 (13.8%) | 0.849 |
| **Neonate-related characteristics** | | | | | | | | |
| Gestational age at birth (weeks) | 29^+4^ [27^+3^, 31^+3^] | 28^+6^ [26^+4^, 30^+5^] | 28^+5^ [26^+5^, 30^+4^] | <0.001^*,**^ | 28^+6^ [27^+1^, 30^+4^] | 28^+6^ [26^+6^, 30^+4^] | 28^+5^ [27^+0^, 30^+3^] | 0.808 |
| Birthweight (g) | 1,200 [950, 1,370] | 1,120 [850, 1.320] | 1,120 [870, 1,320] | <0.001^*,**^ | 1,140 [930, 1,350] | 1,135 [900, 1.330] | 1,123 [900, 1,318] | 0.503 |
| Male gender | 929 (50.3%) | 1,784 (49.8%) | 971 (50.1%) | 0.947 | 357 (50.3%) | 365 (51.4%) | 360 (50.7%) | 0.912 |
| Small for gestational age | 551 (29.8%) | 932 (26.0%) | 431 (22.3%) | <0.001^*,**,†^ | 140 (19.7%) | 128 (18.0%) | 137 (19.3%) | 0.700 |
| Steroid, complete | 815 (59.1%) | 1,709 (60.09%) | 736 (52.3%) | <0.001^**,†^ | 404 (56.9%) | 401 (56.5%) | 412 (58.0%) | 0.830 |
| Delivery room resuscitation | 70 (3.8%) | 157 (4.4%) | 90 (4.7%) | 0.390 | 15 (2.1%) | 15 (2.1%) | 23 (3.2%) | 0.290 |
| Apgar score at 1min | 5 [4, 6] | 5 [3, 6] | 5 [3, 6] | <0.001^*,**^ | 5 [4, 6] | 5 [4, 7] | 5 [3, 6] | 0.023^†^ |
| Apgar score at 5min | 7 [6, 8] | 7 [6, 8] | 7 [6, 8] | <0.001^*,**^ | 7 [6, 8] | 7 [6, 8] | 7 [6, 8] | 0.207 |

Propensity score matching via gestational age, gender, small for gestational age, multiple pregnancies, cesarean section, hypertensive disorders of pregnancy, histologic chorioamnionitis, oligohydramnios, complete course of antenatal corticosteroid use, delivery room resuscitation, and 5 minute Apgar score

Kruskal-Wallis test or chi-square test in both propensity score unmatched population and propensity score matched population

*P<0.05 between low sepsis group and intermediate sepsis group

** P<0.05 between low sepsis group and high sepsis group

†P<0.05 between intermediate sepsis group and high sepsis group

Supplement Table 3. Neonatal outcomes in the study population without infants with early-onset sepsis

|  | Propensity score matching | | | | | | | |
| --- | --- | --- | --- | --- | --- | --- | --- | --- |
|  | Unmatched data set (N=7,363) | | | | Matched data set (N=2,130) | | | |
|  | Low sepsis group  (N=1,847) | Intermediate sepsis group  (N=3,579) | High sepsis group  (N=1,937) | P-value | Low sepsis group  (N=710) | Intermediate sepsis group  (N=710) | High sepsis group  (N=710) | P-value |
| Respiratory distress syndrome | 1,362 (73.7%) | 2,642 (73.8%) | 1,684 (86.9%) | <0.001^**,†^ | 568 (80.0%) | 526 (74.1%) | 619 (87.2%) | <0.001^*,**,†^ |
| Surfactant use | 1,357 (73.5%) | 2,735 (76.4%) | 1,669 (86.2%) | <0.001^**,†^ | 570 (80.3%) | 548 (77.2%) | 616 (86.8%) | <0.001^**,†^ |
| PDA with treatment | 608 (40.9%) | 1,196 (42.1%) | 792 (50.6%) | <0.001^**,†^ | 249 (44.4%) | 227 (41.7%) | 282 (50.1%) | 0.017‡ |
| Hypotension within one week | 295 (16.0%) | 763 (21.3%) | 597 (30.8%) | <0.001^*,**,†^ | 122 (17.2%) | 137 (19.3%) | 207 (29.2%) | <0.001^**,†^ |
| NEC≥stage 2 | 68 (3.7%) | 218 (6.1%) | 156 (8.1%) | <0.001^*,**,†^ | 28 (4.0%) | 43 (6.1%) | 49(7.0%) | 0.044^**^ |
| Isolated intestinal perforation | 12 (0.7%) | 70 (2.0%) | 50 (2.6%) | <0.001^*,**^ | 4 (0.6%) | 16 (2.3%) | 19 (2.7 %) | 0.007^*,**^ |
| IVH≥grade 3 | 87 (4.8%) | 302 (8.7%) | 202 (10.7%) | <0.001^*,**,†^ | 36 (5.1%) | 52 (7.5%) | 75 (10.1%) | <0.001^**^ |
| PVL | 100 (5.5%) | 243 (7.1%) | 177 (9.4%) | <0.001^**,†^ | 43 (6.1%) | 34 (4.9%) | 77 (11.1%) | <0.001^**,†^ |
| Moderate to severe BPD | 379 (22.3%) | 799 (25.2%) | 567 (34.7%) | <0.001^**,†^ | 172 (26.1%) | 160 (24.7%) | 217 (34.9%) | <0.001^**,†^ |
| Severe BPD | 183 (10.8%) | 480 (15.1%) | 346 (21.2%) | <0.001^*,**,†^ | 85 (12.9%) | 94 (14.5%) | 136 (21.9%) | <0.001^**,†^ |
| BPD with steroid use | 203 (11.0%) | 626 (17.5%) | 502 (25.9%) | <0.001^*,**,†^ | 76 (10.7%) | 131 (18.5%) | 177 (24.9%) | <0.001^*,**,†^ |
| Pulmonary hypertension requiring treatment | 73 (4.0%) | 221 (6.2%) | 181 (9.3%) | <0.001^*,**,†^ | 28 (3.9%) | 44 (6.2%) | 67 (9.4%) | <0.001^**^ |
| ROP needs surgery or VEGF | 138 (12.6%) | 329 (14.4%) | 164 (14.6%) | 0.285 | 71 (16.6%) | 59 (13.3%) | 63 (16.1%) | 0.339 |
| Death | 149 (8.1%) | 434 (12.1%) | 326 (16.8%) | <0.001^*,**,†^ | 57 (8.0%) | 68 (9.6%) | 96 (13.5%) | 0.002^**^ |
| Discharge with respiratory support | 255 (15.0%) | 554 (17.6%) | 313 (19.4%) | 0.003^**^ | 111 (17.0%) | 122 (19.0%) | 137 (22.3%) | 0.055 |
| Hospital day | 56 [40, 79] | 58 [38, 84] | 57 [38, 79] | 0.061 | 62 [45, 82] | 58 [40, 83] | 59 [41, 79] | 0.064 |
| Duration of invasive ventilator care | 2 [0, 8] | 3 [0, 17] | 4 [1, 18] | <0.001^*,**,†^ | 2 [0, 8] | 3 [0, 15] | 4 [1, 19] | <0.001^**,†^ |
| Duration of total respiratory support | 19 [5, 45] | 25 [6, 54] | 31 [10, 58] | <0.001^*,**,†^ | 27 [7, 51] | 26 [6, 56] | 32 [11, 58] | <0.001^**,†^ |
| Days to full feeding | 12 [8, 21] | 15 [8, 29] | 19 [10, 35] | <0.001^*,**,†^ | 14 [9, 22] | 15 [8, 27] | 18 [10, 35] | <0.001^**,†^ |
| In survivors | | | | | | | | |
| Hospital day | 58 [43, 80] | 62 [44, 87] | 62 [46, 84] | <0.001^*,**^ | 65 [47, 83] | 60 [44, 85] | 63 [47, 81] | 0.283 |
| Duration of invasive ventilator care | 2 [0, 6] | 2 [0, 14] | 3 [1, 16] | <0.001^*,**,†^ | 2 [0, 7] | 2 [0, 13] | 4 [1, 18] | <0.001^*,**,†^ |
| Duration of total respiratory support | 20 [5, 46] | 28 [6, 56] | 35 [12, 61] | <0.001^*,**,†^ | 28 [7, 52] | 28 [6, 57] | 37 [13, 61] | <0.001^**,†^ |
| Days to full feeding | 12 [8, 21] | 15 [8, 29] | 18 [10, 34] | <0.001^*,**,†^ | 14 [9, 22] | 15 [8, 27] | 18 [10, 35] | <0.001^**,†^ |

Propensity score matching via gestational age, gender, small for gestational age, multiple pregnancies, cesarean section, hypertensive disorders of pregnancy, histologic chorioamnionitis, oligohydramnios, complete course of antenatal corticosteroid use, delivery room resuscitation, and 5 minute Apgar score

Kruskal-Wallis test or chi-square test in both propensity score unmatched population and propensity score matched population

*P<0.05 between low sepsis group and intermediate sepsis group

** P<0.05 between low sepsis group and high sepsis group

†P<0.05 between intermediate sepsis group and high sepsis group

‡ Not statistically significant after Bonferroni correction

PDA; patent ductus arteriosus, BPD; bronchopulmonary dysplasia, IVH; intraventricular hemorrhage, PVL; periventricular leukomalacia, NEC; necrotizing enterocolitis, ROP; retinopathy of prematurity, VEGF; vasculoendothelial growth factor

Supplement Table 4. Logistic regression analysis with random effect after propensity score matching in infants without early-onset sepsis

|  | Univariate logistic regression | | | Multivariate logistic regression  adjusted for gestational age and groups according to the mean annual number of very-low-birthweight infants | | |
| --- | --- | --- | --- | --- | --- | --- |
|  | IS group versus  LS group | HS group versus  IS group | HS group versus  LS group | IS group versus  LS group | HS group versus  IS group | HS group versus  LS group |
| Death | 1.2 (0.84-1.75) | 1.5 (1.06-2.05) | 1.8 (1.27-2.53) | 1.1 (0.71-1.60) | 1.8 (1.25-2.63) | 1.9 (1.32-2.83) |
| Moderate to severe BPD | 1.1 (0.82-1.37) | 1.5 (1.15-1.93) | 1.6 (1.23-2.02) | 1.0 (0.77-1.37) | 1.7 (1.29-2.31) | 1.8 (1.35-2.35) |
| Severe BPD | 1.2 (0.89-1.70) | 1.6 (1.16-2.13) | 1.9 (1.42-2.63) | 1.2 (0.85-1.70) | 1.8 (1.29-2.53) | 2.2 (1.55-3.02) |
| Moderate to severe BPD or death | 1.1 (0.87-1.39) | 1.6 (1.26-2.00) | 1.7 (1.39-2.19) | 1.0 (0.79-1.35) | 1.9 (1.47-2.51) | 2.0 (1.52-2.58) |
| Severe BPD or death | 1.2 (0.95-1.61) | 1.6 (1.27-2.09) | 2.0 (1.56-2.59) | 1.2 (0.87-1.59) | 2.0 (1.50-2.67) | 2.3 (1.75-3.14) |
| PVL | 1.0 (0.64-1.66) | 2.2 (1.44-3.49) | 2.3 (1.53-3.49) | 1.0 (0.65-1.69) | 2.2 (1.43-3.48) | 2.3 (1.53-3.52) |
| Survival without major morbidities* | 1.1 (0.89-1.41) | 0.7 (0.52-0.85) | 0.7 (0.58-0.94) | 1.1 (0.90-1.44) | 0.7 (0.51-0.84) | 0.7 (0.58-0.95) |

*major morbidities include moderate to severe BPD, NEC≥stage 2, IVH≥grade3, PVL, ROP requiring surgery or VEGF

HS; high sepsis, IS; intermediate sepsis, LS; low sepsis, BPD; bronchopulmonary dysplasia, PVL; periventricular leukomalacia
